# Uptake mechanisms and physiological effects of furanic compounds from the Maillard reaction in budding yeast

**DOI:** 10.1002/yea.70013

**Published:** 2026-03-14

**Authors:** Laise Cedraz Pinto Matos, Amy Milburn, Chris MacDonald

**Affiliations:** 1 Department of Food Science, School of Nutrition, Federal University of Bahia (UFBA), Basilio da Gama Street s/n, Canela Campus, 40.110-907, Salvador, Bahia, Brazil; 2 Graduate Program in Food, Nutrition and Health, School of Nutrition, Federal University of Bahia (UFBA), Basilio da Gama Street s/n, Canela Campus, 40.110-907, Salvador, Bahia, Brazil; 3 York Biomedical Research Institute and Department of Biology, University of York, York, UK

**Keywords:** Budding yeast, Maillard reaction products, furanic compounds, transporters, endocytosis, membrane trafficking, toxicity

## Abstract

Maillard reaction products (MRPs) are formed during the thermal processing of foods and exhibit important sensory attributes. Furanic compounds are a subset of MRPs commonly found in food products that are toxic to eukarytoic cells, although the mechanisms of toxicity are poorly understood. We used budding yeast to explore uptake mechanisms of common furanic compounds: 5-hydroxymethylfurfural (HMF), furfural (FUR), and 2-Furyl methyl ketone (FMK). Titrations of each furanic compound were used to identify concentrations that have an inhibitory effect on growth. We identified HMF as a potential substrate of the Pdr5 multidrug resistance pump and linked HMF and FUR toxicity to surface nutrient transporter levels. Live cell imaging shows that HMF disrupts mitochondria whilst FUR affects the endolysosomal system. Results indicate these furanic compounds may have distinct uptake, efflux, and toxicity mechanisms. As many of these cellular components are conserved throughout evolution, this work could shed light on the metabolism of toxic compounds commonly found within animal food sources.

## Introduction

The Maillard reaction is a complex cascade of non-enzymatic browning processes between reducing sugars and amino acids, and is fundamental to the development of flavour, aroma, and colour in thermally processed foods (Liu et al., 2022). The array of molecules produced, collectively termed as Maillard reaction products (MRPs), have distinct sensory implications for the food industry (Hosry et al., 2025; Tamanna & Mahmood, 2015). The interplay of various factors, such as chemical composition, temperature, time, humidity, and pH, along with the presence of glycation agents or oxidants, dictates the progression of this chemical cascade and the specific MRPs generated (Nie et al., 2013; Shakoor et al., 2022). Various MRPs have been identified in foods, common examples include acrylamide and furanic compounds (Agcam, 2022; Sun et al., 2022). Humans are frequently exposed to furanic compounds, such as 5-hydroxymethylfurfural (HMF), furfural (FUR), and 2-Furyl methyl ketone (FMK), which are associated with sugar-rich foods (National Toxicology Program, 2010). However, beyond sensory appeal, this reaction also generates a broad spectrum of potentially harmful by-products (Kathuria et al., 2023).

The toxicity of MRPs has been extensively debated, with examples like acrylamide being correlated with various diseases and negative effects (Başaran et al., 2023). However, biological effects of MRPs are context-dependent, varying according to factors like dose, stability, biological model, and metabolic capacity, (Cheng et al., 2022; Wierckx et al., 2011). Furanic compounds are known to be carcinogenic (National Toxicology Program, 1993). Studies using rodent species have shown that ingestion of furanic compounds induces DNA damage on spleen/liver cells, triggering chromosomal aberrations and cell death (Leopardi et al., 2010; Neuwirth et al., 2012; Yilmaz et al., 2023). In addition to genotoxic effects, furanic compounds are known to induce oxidative stress and inflammation (EFSA Panel et al., 2017). For example, HMF increases reactive oxygen species (ROS) in both fly larvae and cultured human cell models, with the latter example showing ROS triggers apoptosis via mitochondrial pathways (Chen et al., 2024; Qiu et al., 2022). Furanic compounds are known to be produced in substrates used for microorganism conversion to biofuel, which hinders the fermentation process (Wang et al., 2016). In *Saccharomyces cerevisiae*, exposure to these compounds is toxic and leads to elevated ROS levels (Allen et al., 2010). This oxidative stress response may be linked to mitochondrial dysfunction, as directed-evolution approaches aimed at increasing furanic tolerance consistently recovered mutations affecting mitochondrial pathways (Ren et al., 2024).

MRPs are present in food in free form or conjugated to proteins, peptides or polyphenols (Nowak et al., 2021; Zhu et al., 2020). The health effects of these compounds are primarily evaluated in isolation, as MRPs are readily absorbed in the gastrointestinal tract and thus these forms represent the bioavailable fraction (Pagare et al., 2024). Regulatory agencies use measurements of compounds such as HMF, acrylamide and furfurals in free form to estimate dietary exposure, construct safety limits and compare with the acceptable daily intake (EFSA_Panel et al., 2017; Eisenbrand, 2020). The ready conversion of bound to free MRP compounds after acid and enzymatic digestion has already been demonstrated in cells (Zeng et al., 2020). Furthermore, the use of isolated analytical standards also allows the evaluation of toxicity and cellular mechanisms with standardized and reproducible protocols (Qiu et al., 2022), meeting the requirements of toxicological evaluation. Thus, the free form of MRPs is more directly related to acute systemic effects, such as cytotoxicity, genotoxicity, and metabolic alterations, which are biologically more relevant.

Despite extensive toxicological characterisation of furanic aldehydes, a longstanding consensus in the literature is that small furan derivatives, such as HMF, FUR, and related molecules, enter eukaryotic cells predominantly by passive diffusion across the lipid bilayer. This view is grounded in their low molecular weight, moderate polarity, and aromaticity, all of which favour membrane permeability without requiring carrier proteins (Heer & Sauer, 2008; Klinke et al., 2004). Consequently, passive diffusion has been widely accepted as the default explanation for cellular uptake of these compounds, even though it does not exclude the possibility that transporter systems modulate their intracellular retention or contribute to compound-specific toxicity. Other studies have challenged the general assumption that xenobiotics freely cross membranes, arguing instead that many small molecules previously considered to be passively permeable are in fact subject to transporter-mediated flux in eukaryotic cells (Baril et al., 2023; Kell, 2021). Revisiting this debate is therefore essential for understanding how furanic inhibitors interact with conserved eukaryotic pathways of stress response, detoxification, and organellar homeostasis.

Eukaryotic cells adjust their uptake and export of nutrients in response to environmental conditions. *Saccharomyces cerevisiae* is a powerful platform for dissecting these mechanisms at the molecular level. Yeast employs a broad array of surface transporters for amino acids, sugars, and metal ions (Bianchi et al., 2019; Donzella et al., 2023). Yeast surface proteins are routinely internalized to endosomes and follow conserved recycling pathways back to the plasma membrane (Laidlaw et al., 2022; MacDonald & Piper, 2016, 2017). Alternatively, internalized surface proteins can be ubiquitinated and sent through the Endosomal Sorting Complexes Required for Transport (ESCRT) mediated pathway to the yeast lysosome (or vacuole) for degradation (Laidlaw & MacDonald, 2018). This downregulation can be triggered in response to substrate or stress, and is mediated upstream of ESCRTs by the arrestin related trafficking adaptors, which specifically unite transporters with the E3 ubiquitin ligase Rsp5 to mediate ubiquitination (C. H. Lin et al., 2008; MacDonald et al., 2020; Nikko et al., 2008). Beyond these traditional nutrient uptake mechanisms, yeast also express multidrug resistance transporter proteins, that transport various substances and toxic compounds, out of the cell (Piecuch & Obłąk, 2014). Amongst these, the Pdr5 multidrug transporter is the best characterized, which has remarkable promiscuity for diverse substrates, and is therefore of interest in clinical and biotechnological applications (Golin & Ambudkar, 2015; Rogers et al., 2001). Whether furanic compounds are substrates of any of these transporters, in yeast or other eukaryotes, has not been directly tested and is not well understood. However, recent transcriptome analyses did suggest expression of pumps like Pdr5 might correlate with toxicity of furanic compounds added to anaerobic yeast cultures of a xylose-utilizing yeast strain (Ask et al., 2013)

Functional aspects of surface transporters are conserved between yeast and mammalian cells (Belle & André, 2001). Additionally, transporter regulatory mechanisms, such as ubiquitin mediated degradation, engagement with alpha arrestins, and endolysosomal trafficking are also evolutionarily conserved (Alvarez, 2008; Laidlaw & MacDonald, 2018; Piper & Katzmann, 2007). In this study, we hypothesise furanic compounds are actively transported across the plasma membrane, and that uptake and downstream cellular effects could be evolutionarily conserved. Using yeast, we use different genetic systems to explore transporter regulation of furanic compounds, and cell biological approaches to assess disruption of cellular processes. This work sets out to define fundamental mechanisms contributing to furanic-induced toxicity and the physiological responses of eukaryotic cells, ultimately contributing to a broader understanding of food-derived toxicants and their impact on human health.

## Results

### MRPs induce toxic effects in yeast cells

**Figure 1:**
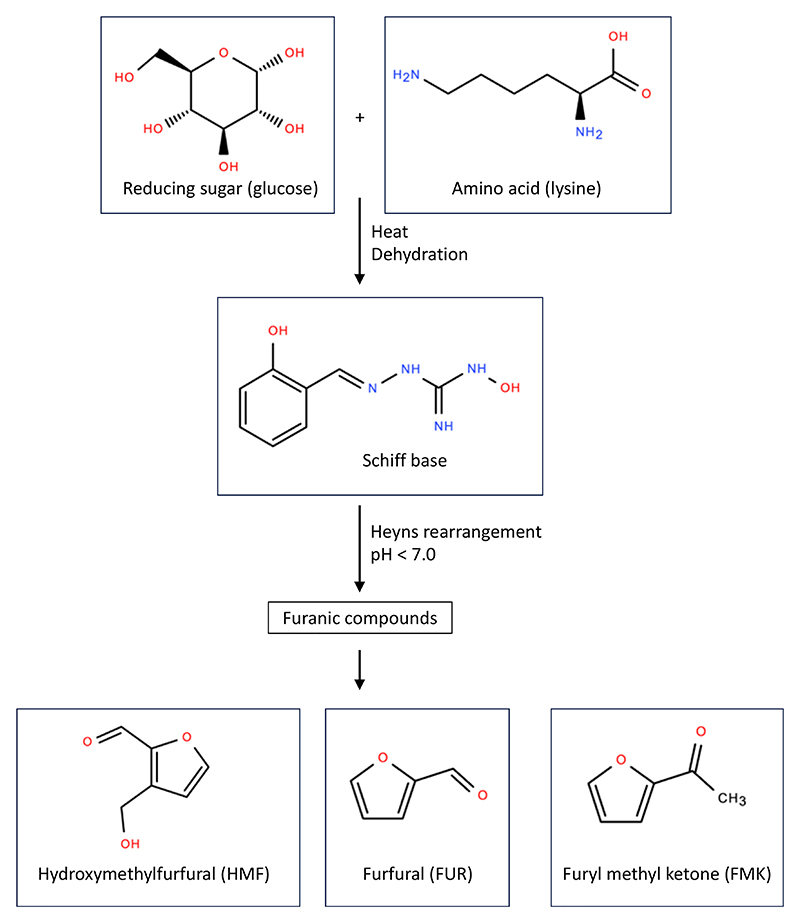
Chemical cascade of the Maillard reaction The non-enzymatic reaction between carbonyl groups of reducing sugars and an amine group triggers a cascade with many products. Those specific to the production of furfurals are depicted.

**Figure 2:**
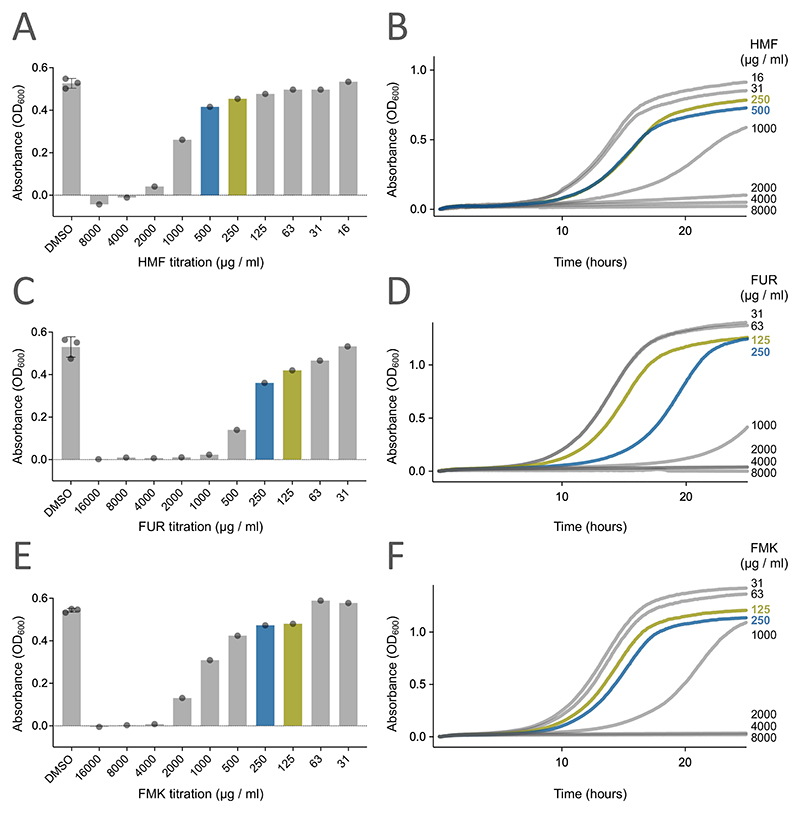
Furanic compounds exert toxic effects on yeast cells **A, C, E)** Endpoint assays were performed with wild-type cells were exposed to varying concentrations of Hydroxymethylfurfural (HMF), Furfural (FUR) and 2-Furyl methyl ketone (FMK). The OD_600_ values were measured of 200 μl cultures grown in 96-well plates immediately after inoculation and then again following 24 hours growth at 30°C. **B, D, F)** Yeast growth assays across a titration of HMF, FUR and FMK were performed in liquid cultures with continuous measurements of OD_600_ every 5 minutes. In each experiment, concentrations of furanic compounds which are used for downstream experiments are indicated (blue and green).

To determine if the budding yeast *Saccharomyces cerevisiae* responded to different furanic compounds, we selected three representatives commonly found in food: HMF, FUR and FMK (Figure 1). For initial toxicity experiments, we optimised a 96-well plate end-point growth assay, where cell density at the start of the experiment was compared to later time points. Using this assay, we tested titrations of HMF, FUR and FMK across ranges predicted to exert a physiological effect, based on previous studies in mammalian cells (Kong et al., 2019; Qiu et al., 2022; Zhao et al., 2017). These experiments showed that yeast exposed to HMF at concentrations of 125 μg/ml or less had no significant defect in growth after 24 hours (Figure 2A). However, at higher HMF concentrations, growth was inhibited in a concentration dependent manner. To validate these results, we employed our recently optimised continuous measurement growth assay and analysis pipeline (Laidlaw et al., 2025), which also showed a HMF concentration dependent inhibition of yeast growth, with reduced growth efficiency observed for 250 μg/ml and above, with almost complete growth inhibition at concentrations of 2000 μg/ml or higher (Figure 2B). We performed similar analysis with other MRPs. Both assays revealed a concentration dependent effect on yeast growth in media containing FUR, with significant growth inhibition at concentrations of 125 μg/mL and above (Figures 2C - 2D). Similarly, titrations of FMK showed a range of growth effects, with inhibition in the range 125-250 μg/mL and more potent effects at concentrations of 500 μg/mL and above (Figures 2E - 2F).

**Figure 3:**
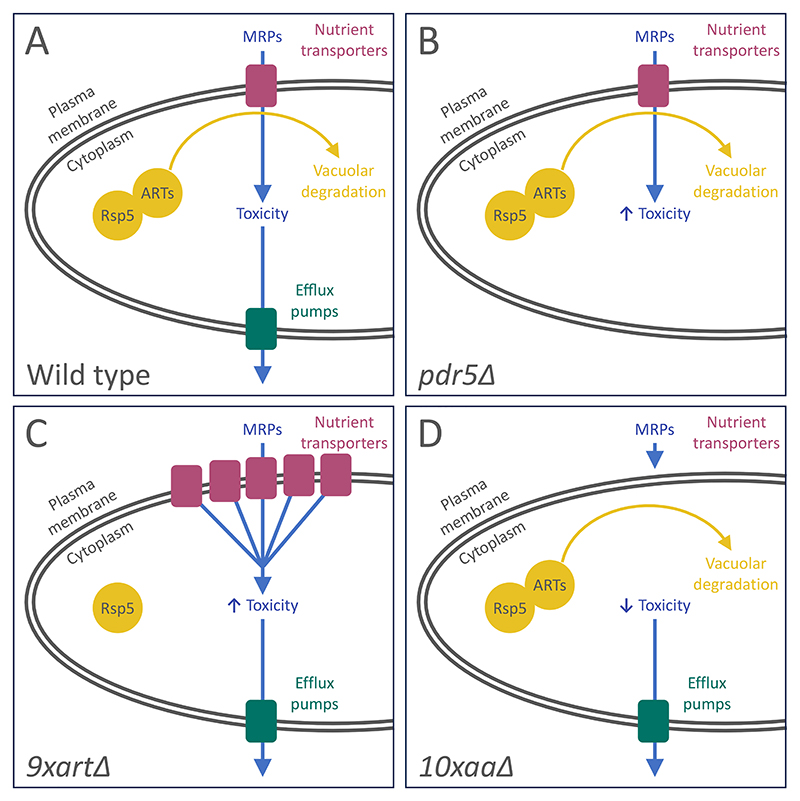
Genetic systems to test sensitivity of furanic compounds in yeast **(A)** Wild-type yeast expressing nutrient transporters uptake a range of nutrients across the plasma membrane, with toxic compounds being driven out of the cell through the action of efflux pumps like Pdr5. **B)** If harmful compounds, such as furanic compounds, are substrates of the Pdr5-efflux pump, an increase in toxicity would be predicted in *pdr5*Δ deletion mutants. **C)** Nutrient transporters are routinely downregulated by the vacuolar degradation pathway, where the E3 ubiquitin ligase Rsp5 is recruited to specific transporters via ART adaptors to ubiquitinate and trigger their degradation. A strain lacking nine of these ART adaptors (*9xart*Δ) has higher levels of different transporters at the plasma membrane, which we hypothesize would elevate toxicity via uptake of any compounds by transporters. **D)** Conversely, if toxic compounds rely on transporters for uptake into the cell, a strain lacking many different amino acid transporters (*10xaa*Δ) would be protected from the effects.

Collectively, this work shows that all three compounds from the Maillard reaction have toxic effects on yeast growth. We therefore set out to use the yeast system to test the hypothesis that transporters influence the cellular uptake and resulting intracellular accumulation of furanic compounds, which are related to common transporter substrates, like sugars and amino acids (Ask et al., 2013; Cong et al., 2021). We reasoned any such non-canonical flux via transporters would not be specific, so sought genetic approaches broadly affecting pathways, instead of specific transporters, to test this hypothesis (Figure 3).

### The Pdr5 efflux pump selectively regulates furanic compounds

**Figure 4:**
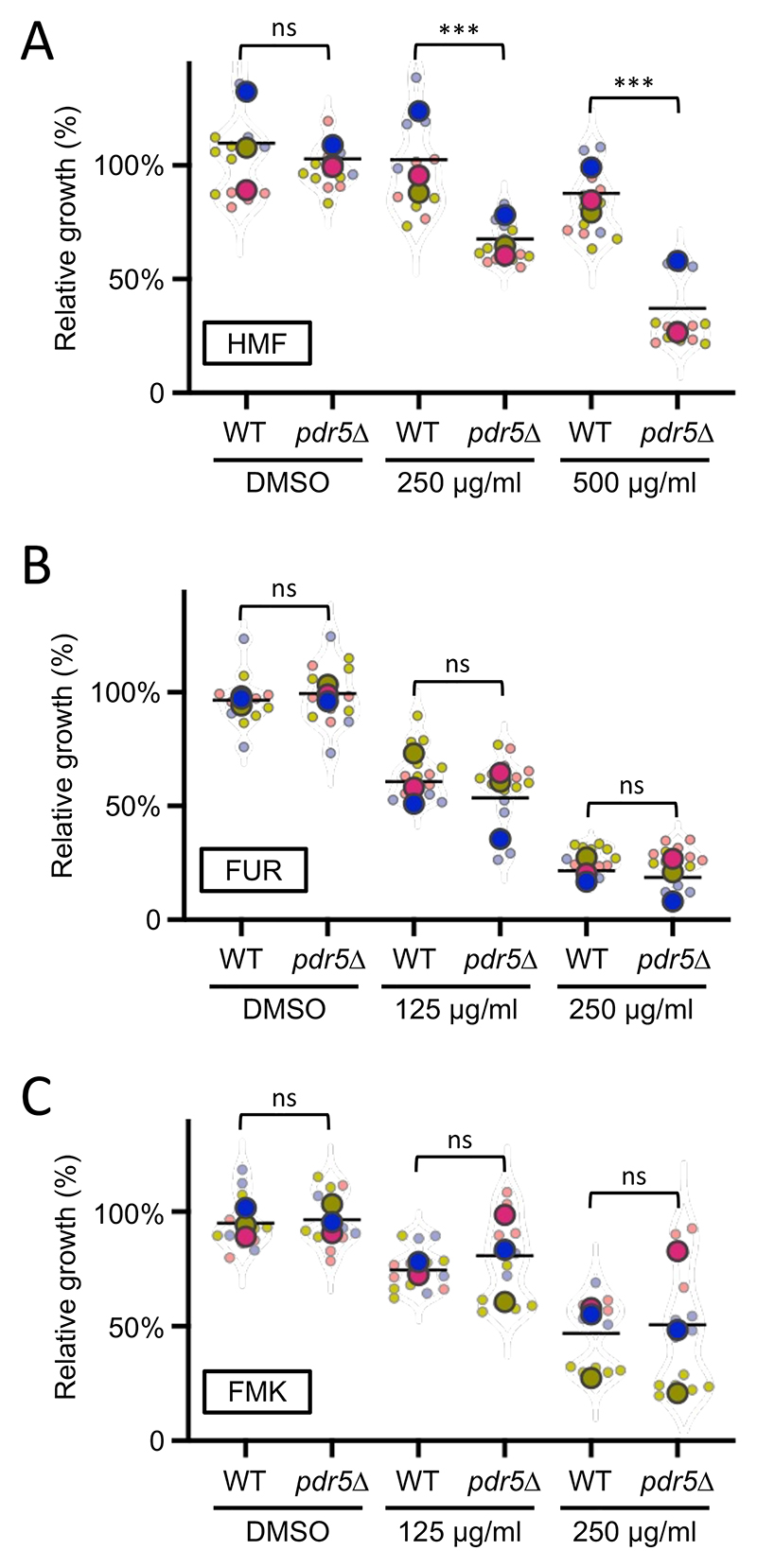
*pdr5*Δ mutants are hypersensitive to exposure of HMF **A, B, C)** Relative growth of wild-type (WT) and *pdr5*Δ cells was assessed following exposure to furanic compounds, with DMSO as a control. Cultures were used to inoculate media containing indicated concentrations of HMF **(A)**, FUR **(B)**, and FMK **(C)** followed by growth measurements after 24 hours at room temperature. One-Way ANOVA and Sidak’s multiple comparisons tests were performed. (ns) not significant, (***) p<0.001.

Pdr5 is a multidrug surface transporter that drives efflux of many toxic molecules to confer resistance (Balzi et al., 1994; Leppert et al., 1990). We reasoned that the harmful effects of high doses of different furanic compounds could be ameliorated by Pdr5, predicting *pdr5*Δ cells would be hypersensitive to MRPs (Figure 3B). To test this hypothesis, we compared growth of wild-type yeast and *pdr5*Δ deletion mutants in the presence of previously identified toxic concentrations of HMF, FUR and FMK. Although wild-type and *pdr5*Δ mutants have no significant growth difference in DMSO control media, we found *pdr5*Δ cells are hypersensitive to HMF, growing to only 68% ± 9 of wild-type cells in the presence of 250 μg/mL HMF (Figure 4A). At the higher concentration of 500 μg/mL HMF, wild-type cells grow to 88% ± 10 but *pdr5*Δ cells 37% ± 7. Experiments with FUR at concentrations that inhibit growth showed no significant difference between wild-type cells and mutants with *PDR5* deleted (Figures 4B). 125 μg/mL FUR reduced growth of wild-type cells and *pdr5*Δ cells to 60% ± 11 and 54% ± 16, respectively. 250 μg/mL FUR did exert a more potent effect in wild-type cells 21% ± 5, but was very similar to *pdr5*Δ cells that retained only 19% ± 10. In much the same way, both 125 and 250 μg/mL FMK inhibited growth of wild-type and *pdr5*Δ cells in a similar, albeit concentration dependent manner (Figure 4C). We speculate that wild-type cells might efflux HMF via Pdr5, resulting in the growth benefit of wild-type cells compared to *pdr5*Δ mutants. These results further suggest that each furanic compound affects yeast cells through a different mechanism: HMF appears to be actively effluxed by Pdr5 at toxic concentrations, whereas FUR and FMK show no evidence of Pdr5-dependent transport. Therefore, the cellular response to these compounds is not uniform but compound-specific. Internal control experiments were included in all experiments, showing that wild-type and *pdr5*Δ strains reached a similar final cell density in media treated with DMSO alone.

### Modulation of surface transporters correlates with furanic compound sensitivity

**Figure 5:**
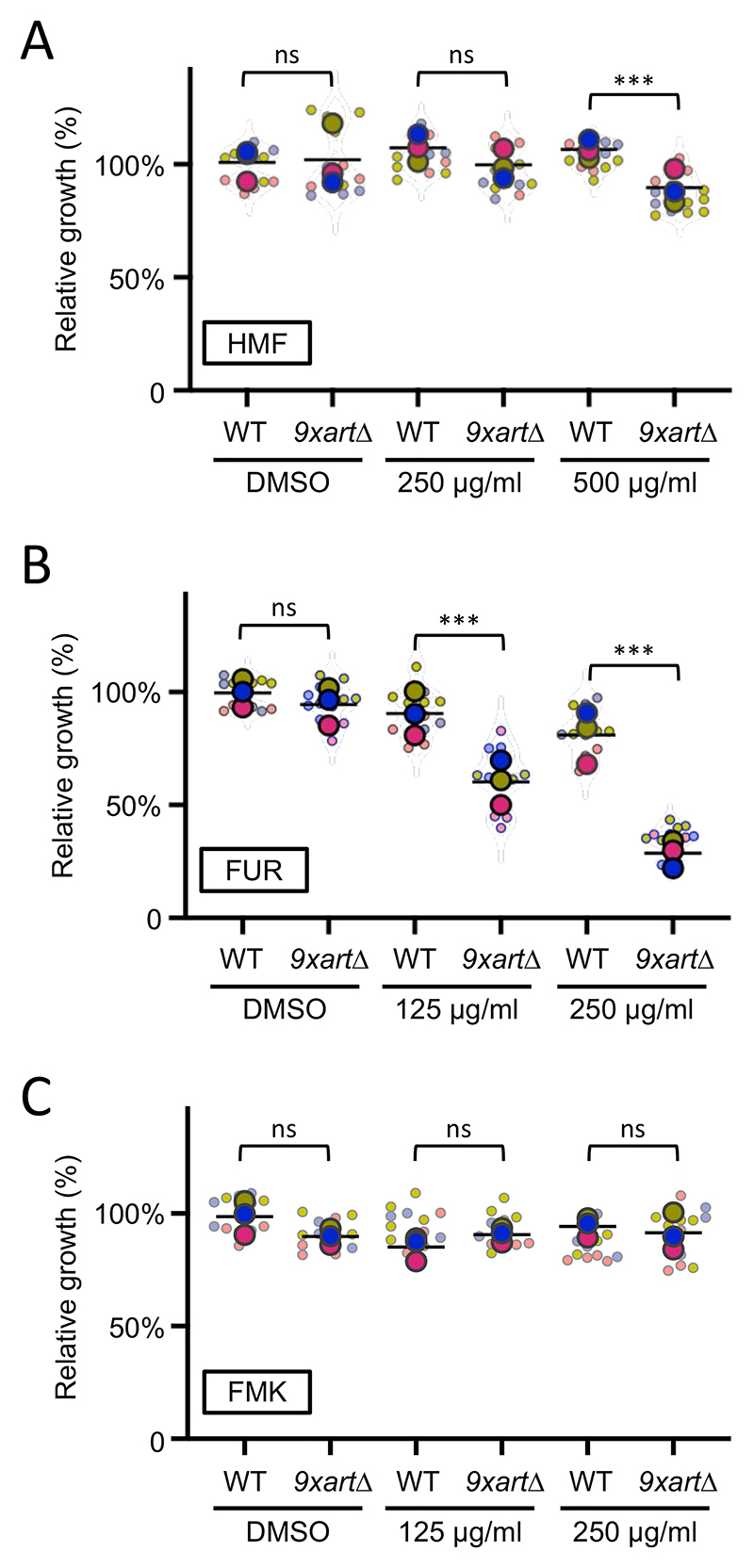
Perturbation of endosomal trafficking adaptors sensitizes cells to furanic compounds **A, B, C)** End point growth assays were used to compare growth in DMSO controls or following exposure to furanic compounds: HMF **(A)**, FUR **(B)**, and FMK **(C)** after 48 hours. Wild-type cells were compared to a *9xart*Δ mutant strain across all conditions. One-Way ANOVA and Sidak’s multiple comparisons tests are indicated: (ns) not significant, (***) p<0.001.

As furanic compounds are relatively similar to natural substrates of yeast transporters, such as amino acids, we considered that nutrient transporters might uptake furanic compounds. To broadly test this idea, we employed a haploid mutant strain lacking 9 different alpha-arrestin (*9xart*Δ) or arrestin-related trafficking (ART) adaptors (Nikko & Pelham, 2009). These proteins act to bridge an array of nutrient transporters with the promiscuous E3 ubiquitin ligase, Rsp5 (Kahlhofer et al., 2021). Deletion of arrestins therefore elevate surface levels of nutrient transporters, allowing increased uptake of exogenous molecules, potentially including furanic compounds (Figure 3C). There was almost no difference in growth between wild-type and *9xart*Δ cells upon addition of HMF after 48 hours at either concentration, besides a very subtle hypersensitive phenotype at 500 μg/ml HMF in *9xart*Δ (Figure 5A). Much more strikingly, the *9xart*Δ strain was hypersensitive to FUR, with concentration dependent growth inhibition at both 250 and 500 μg/mL concentrations (Figure 5B). Similar to HMF, different doses of FMK were equally inhibitory between wild-type and *9xart*Δ strains (Figure 5C). These data suggest FUR, and potentially HMF when administered at higher doses, may require surface localisation of nutrient transporters to exert toxic effects on yeast cells.

**Figure 6:**
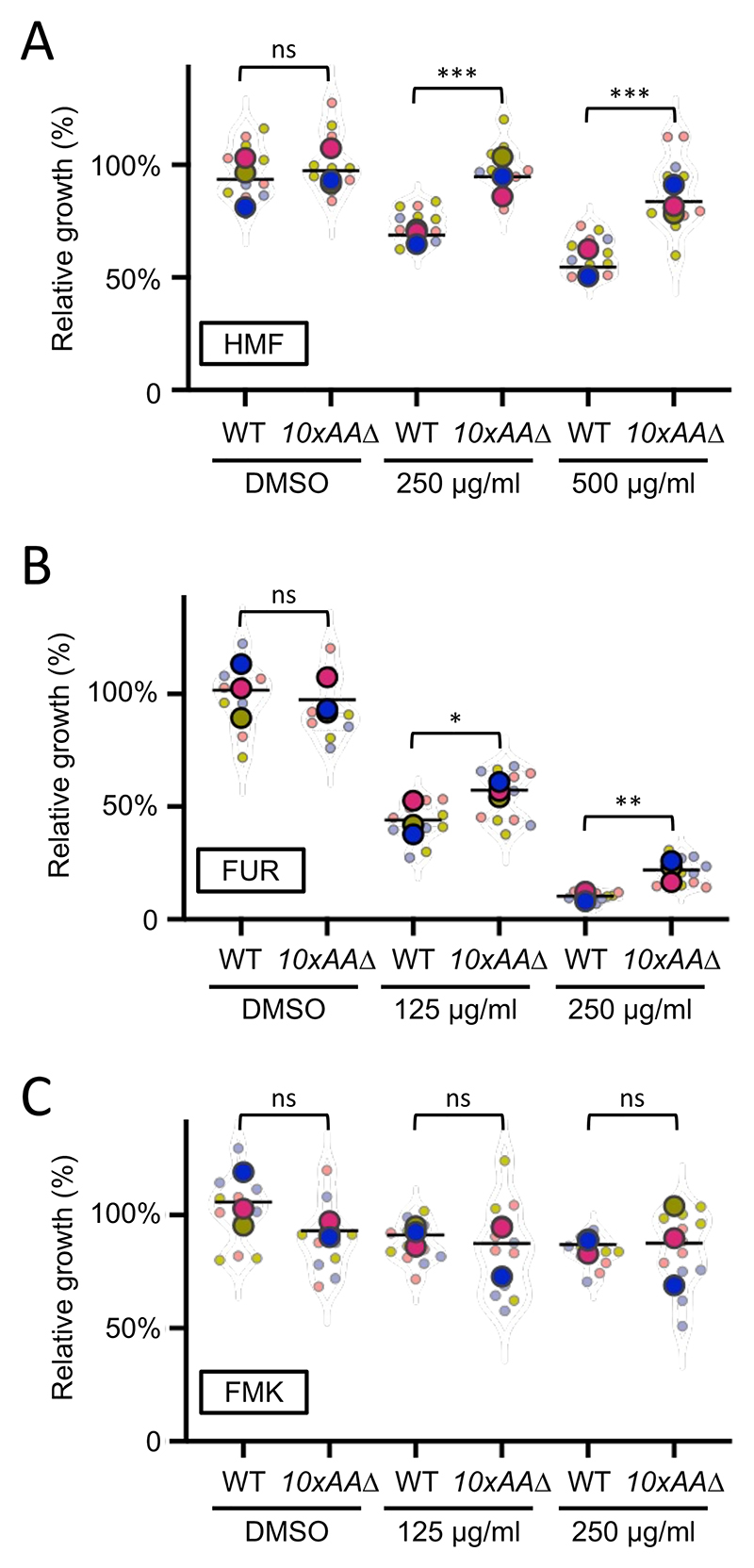
Cells lacking nutrient transporters tolerate furanic compounds **A, B, C)** Relative growth of wild-type (WT) cells was compared to a mutant lacking 10 different amnio acid transporters (*10xaa*Δ). Cells were exposed to HMF **(A)**, FUR **(B)**, and FMK **(C)**, with DMSO added as a control for 24 hours porior to measurements. One-Way ANOVA and Sidak’s multiple comparisons tests were performed. (ns) not significant, (*) p<0.05, (**) p<0.01, (***) p<0.001.

To further investigate the hypothesis that nutrient transporter retention at the plasma membrane drives MRP-induced hypersensitivity in *9xart*Δ cells, we pursued a complementary experimental strategy. Given that arrestin deletion stabilizes transporters at the cell surface, we reasoned that eliminating the transporters themselves would yield the opposite phenotype (Figure 3D). To test this, we used the *10xaa*Δ strain; this strain lacks 10 distinct nutrient transporters and is severely deficient in the uptake of most proteinogenic amino acids (Besnard et al., 2016). These experiments revealed that the *10xaa*Δ strain is more resistant to HMF and FUR than wild-type cells (Figure 6A - 6B), but reaches a similar final cell density as wild-type cells under DMSO control conditions. This pattern is consistent with the possibility that HMF and FUR exert reduced inhibitory effects when major surface nutrient transporters are absent. This interpretation is further supported by the observation that these compounds elicit hypersensitive phenotypes in mutants with elevated surface transporter abundance (Figure 5). Furthermore, FMK, which had no arrestin-related phenotype, also does not have any significant changes in effects in wild-type cells compared with the *10xaa*Δ strain (Figure 6C). Taken together, these observations suggest that, within the conditions tested, furanic compounds are actively uptake and processed in a selective manner by nutrient transporters at the yeast plasma membrane.

### Furanic compounds perturb distinct cellular processes

**Figure 7:**
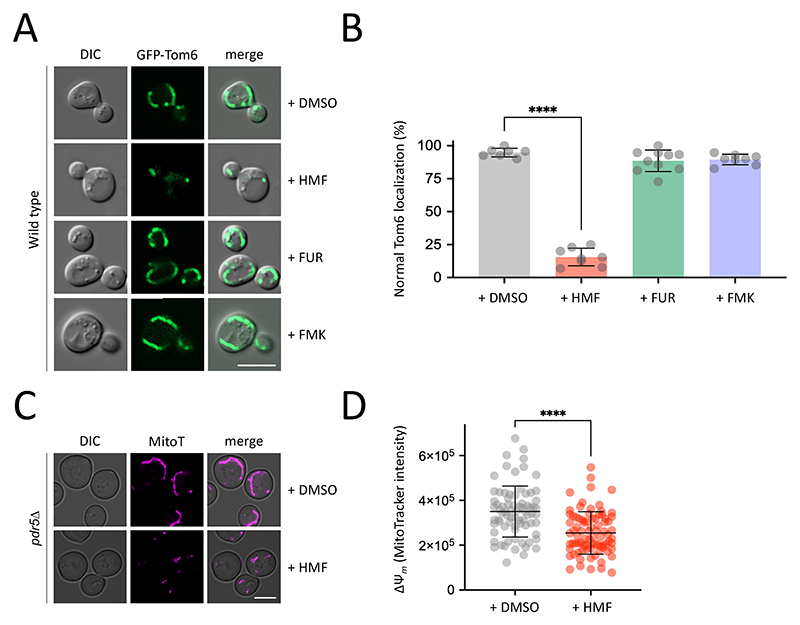
HMF exposure induces mitochondrial defects **A)** Wild-type cells expressing a GFP tagged version of Tom6 (GFP-Tom6) were grown to mid-log phase and imaged by fluorescence microscopy following 4 hours exposure to HMF, FUR, FMK and DMSO as a control. **B)** The average number of cells (n = 150 - 200, per condition) across replicates (n > 6) that exhibit defective mitochondrial morphology are presented. **C)** Mid-log *pdr5*Δ cells were grown to mid-log phase, treated with DMSO or 2000 μg/ml HMF for 4 hours prior to labelling of mitochondria using 2 μM MitoTracker CMXRos. Cells were imaged using laser scanning confocal microscopy. **D)** The fluorescence intensity of MitoTracker stained cells from (C) was measured as an indirect measure of mitochondrial membrane potential (ΔΨ_*m*_), with n > 75 cells measured per condition. (****) p < 0.0001 shown by unpaired Holm–Sidak *t*-test. Scale bars: 5 μm.

We show that yeast can be used to study MRP effects on eukaryotic cells, and that MRPs like HMF, FUR, and FMK appear to exert their effects through distinct mechanisms. To support this idea, we surveyed broad biological processes through organelle maintenance in response to furanic compounds, to indicate if specific pathways were involved in recognising or responding to these MRPs. Firstly, as the Maillard reaction is associated with oxidative stress and mitochondrial dysfunction (Allen et al., 2010; Kim & Hahn, 2013; Qiu et al., 2022), we assessed mitochondrial morphology using a GFP tagged version of Tom6, which localises to a ribbon morphology in wild-type cells (Shaw & Nunnari, 2002). We observed a striking defect in mitochondrial morphology following exposure to HMF for 4 hours, with GFP signal restricted to small bright puncta within the cell (Figure 7A). Several mis-localisation patterns of GFP-Tom6 were documented in the presence of HMF, which were absent from DMSO control treatments (Figure S1A). Mitochondria span large regions of the yeast cells, so we performed 3D confocal microscopy to better appreciate the morphology in control cells, which show contiguous ribbons (Supplemental Movie S1). 3D imaging of the same GFP-Tom6 expressing cells treated with HMF revealed punctate localisations spread throughout the cytoplasm of the cell (Supplemental Movie S2). We quantified cells across treatments as a percentage of the total population that exhibit normal, ribbon like mitochondrial structures, with only HMF exhibiting a significant defective morphology phenotype (Figure 7B). We then combined HMF treatment with the hypersensitive *pdr5*Δ strain and assessed mitochondrial membrane potential (ΔΨ_*m*_) using MitoTracker Red CMXRos (Poot et al., 1996). These stained mitochondria also showed fragmentation following HMF treatment, suggesting GFP-Tom6 was a faithful representation of mitochondria (Figure 7C). The signal intensity of MitoTracker was used as a proxy for ΔΨ_*m*_, to show a significant decrease following HMF treatment, suggesting impaired mitochondrial function (Figure 7D).

**Figure 8:**
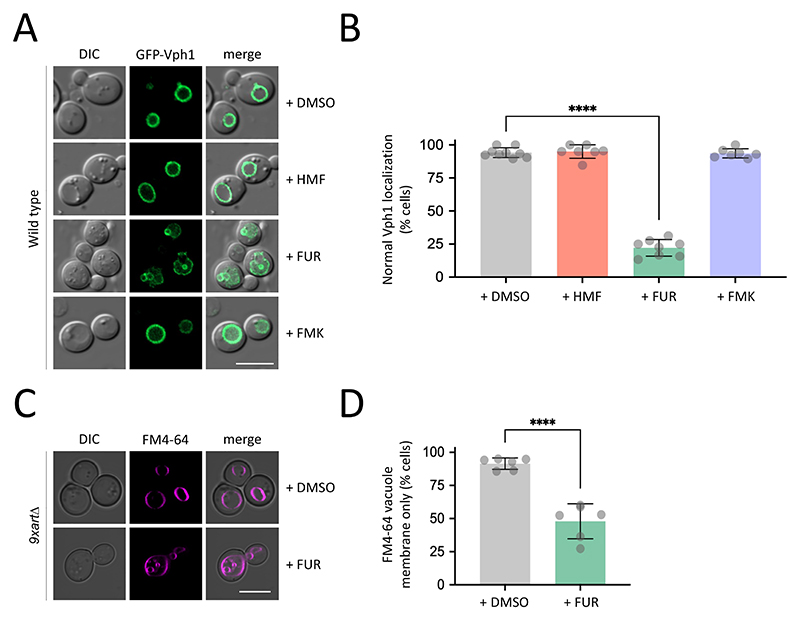
Vacuolar morphology is perturbed following exposure to FUR **A)** Cells expressing GFP-Vph1 were grown to mid-log phase prior to DMSO or furanic compounds (HMF, FUR and FMK) being added to the media for 4 hours prior to confocal microscopy. **B)** The average number of cells (n > 150 per condition) across replicates (n > 5) that exhibit abnormal vacuolar localisations are presented in the histogram as a percentage. **C)** *9xart*Δ cells were grown to log phase prior to 4-hour exposure 1000 μg/ml FUR, with control cells treated with DMSO for the same period. Cells were then labelled with 0.8 μM FM4-64 for 30 minutes, followed by a 1-hour chase period in label free media. Pulse chase steps in the protocol were performed with DMSO and FUR supplemented media. Labelled vacuoles were then imaged by fluorescence microscopy. **D)** Cells from (C) were quantified from experiments (n > 5) and the percentage of cells exhibiting only FM4-64 labelling at the limiting membrane was calculated, compared to cells that were observed to also have intravacuolar signal. (****) p < 0.0001 as determined by unpaired Holm–Sidak *t*-test comparing treatments to DMSO control. Scale bars: 5 μm.

Secondly, as our genetic perturbation of nutrient transporters and arrestins correlated with sensitivity to furanic compounds, we considered the endolysosomal system as a potential target organelle. We chose the vacuole as a representative marker, as it is also known to respond to different stress conditions (Kohler & Büttner, 2021; Li & Kane, 2009). Cells expressing a GFP tagged version of the vacuolar ATPase subunit Vph1 were grown to mid-log phase and then exposed to inhibitory concentrations of different furanic compounds for 4 hours. As expected, the vacuoles of wild-type cells exposed to DMSO showed no defects, which was also true of HMF or FMK treatments (Figure 8A). However, FUR induced a variety of abnormal phenotypes never observed in control cells, including significant GFP-Vph1 inside the lumen of the vacuole, in addition to accumulations at the periphery, alongside other non-vacuolar signal (Figure S1B). Cells were quantified as a percentage exhibiting normal GFP-Vph1 localisations, with FUR treatment the only condition that caused a significant number of cells with abnormal localisations (Figure 8B). This vacuolar disruption phenotype was corroborated in hypersensitive *9xart*Δ cells using the lipid dye FM4-64 following a pulse-chase protocol to label the vacuole, with cells treated with either DMSO or FUR (Figure 8C, S1C). Again, FUR induced a significant percentage of cells with an obvious intralumenal signal of FM4-64 compared to DMSO treatment, where the signal is exclusively localised to the limiting membrane of the vacuole (Figure 8D).

This physiological analysis further supports the idea that individual MRPs have cell specific responses and sensitivities in eukaryotic cells and that many layers of regulation are likely involved in any cellular response to even individual compounds.

## Discussion

The intermediate Maillard reaction products classed as furanic compounds are chemical contaminants created during industrial processes or cooking, commonly identified in food following excessive heating (e.g. honey and caramelised syrups) or on the surface of fried or baked foods (Alsafra et al., 2022; Conceição et al., 2024; Santos et al., 2025; Shapla et al., 2018; Sun et al., 2022). These compounds can form through sugar degradation, lipid peroxidation, or thermal decomposition of amino acids, indicating their diverse origins and potential variability in concentration depending on food matrix and processing method (Hosry et al., 2025). EFSA CONTAM Panel identified a worrying range of furan exposure, particularly consumption of ready-to-eat meals and cereals for infants and coffee for adults (EFSA Panel et al., 2017). Given their presence in foods of habitual consumption and their constant exposure in the human diet, it is crucial to evaluate the dietary exposure to these compounds and to understand the mechanisms associated with their uptake, efflux, and impact on processes at the cellular level. Several studies have also highlighted the potential for furanic compounds to induce oxidative stress and mitochondrial dysfunction, which can contribute to cytotoxicity and metabolic disruption in exposed cells (Allen et al., 2010; Aydin et al., 2023; Batool et al., 2021, 2025; Zhang et al., 2015). In this study we focussed on three furanic products from the Maillard reaction commonly found in food: HMF, FUR, and FMK, which present different functional groups, such as hydroxyl, methyl, and ketone, in the molecular rearrangement linked to the structure of a furan ring (Figure 1). Although these compounds share molecular features, it is important to consider their differences, which might influence cytotoxic potential, mechanisms and specificity of transport in/out of cells, and mode of action that perturbs cellular processes. Moreover, differential interactions with membrane transporters and detoxification enzymes may underlie the distinct cellular responses observed for each compound, emphasising the need for compound-specific toxicokinetic studies (Quan et al., 2022). Understanding these nuances is key to assessing the risk associated with dietary intake of Maillard reaction products and developing strategies to mitigate their impact on human health.

Although previous studies have reported toxic effects of furanic compounds on the growth of eukaryotic cells, including yeast (Ask et al., 2013; Lam et al., 2021), our documentation of a dose response inhibition on growth in response to HMF, FUR and FMK suggests regulatory mechanisms are triggered by this stress, and provide optimised concentrations to explore these physiological responses. As mentioned, although MRPs are complex and often conjugated to other molecules, studying MRPs in isolation best represents the common products encountered by cells (Pagare et al., 2024). Curiously only HMF showed clear phenotypes suggesting clearance via the Pdr5 multidrug resistance pump, with *pdr5*Δ mutants being hypersensitive to different concentrations (Figure 4). This specificity may be related to the higher polarity or redox activity of HMF compared to FUR and FMK, which could enhance recognition or affinity to the substrate-binding cavity for efficient Pdr5 efflux (Egner et al., 1998). As a fungal protein, much of the work on Pdr5 is focussed on biomedical and agricultural applications (Golin & Schmitt, 2023). However, this family of ATP-binding cassette (ABC) proteins are conserved from bacteria to humans (Balzi et al., 1994), with various human homologues shown to transport a range of structurally and functionally distinct substrates across the plasma membrane (Lin & Yamazaki, 2002; Sodani et al., 2012). Therefore, we suggest that multidrug resistance pumps are worthy of consideration for regulation of MRPs consumed in human diets.

We do note that the genetic systems we have used to understand the tolerance of yeast to furanic compounds rely on indirect assumptions. It is possible that, although we know more/fewer nutrient transporters localise and function at the plasma membrane in *9xart*Δ and *10xaa*Δ cells, respectively (Besnard et al., 2016; C. H. Lin et al., 2008; Nikko et al., 2008; Nikko & Pelham, 2009). Alternative explanations could be through the intracellular targets of furanic compounds being mis-localised in these mutant cells, or other indirect consequences, such as membrane destabilisation under stress (Teixeira et al., 2008). However, given results such as *9xart*Δ cells are hypersensitive to FUR and *10xaa*Δ mutants are resistant, we believe the most logical explanation is transport via a surface localised transporter facilitating cellular entry of FUR. To extend this further, we assume at least one of the 10 transporters deleted in the *10xaa*Δ strain has elevated surface retention in the *9xart*Δ strain. Although we acknowledge the nature of what transporter(s) are responsible for these responses is not yet known, and yeast transporters are known to vary in their substrate specificity (Bianchi et al., 2019). Furthermore, environmental pressure can modify yeast transporters, either modulating specificity or creating new activity (Karapanagioti et al., 2024). Therefore, it is reasonable to assume that these furanic compounds, which are chemically similar to natural yeast transporter substrates, might utilise transporter activity to enter cells. Changes in exogenous substrate levels, such as glucose or nitrogen depletion, indirectly regulates surface transporters via post-translational modification of arrestins (Kahlhofer et al., 2021; Megarioti et al., 2021; O’Donnell & Schmidt, 2019). Therefore, as proteins like amino acid transporters and alpha arrestins are highly conserved with an array of human orthologues that perform analogous functions in animal cells (Gauthier-Coles et al., 2021; Zbieralski & Wawrzycka, 2022), these uptake mechanisms of furanic compounds may be conserved in other eukaryotic systems.

Our transporter-based phenotypes provide a mechanistic counterpoint to the longstanding assumption that furanic aldehydes enter cells exclusively by passive diffusion. This view, rooted in classical fermentation literature, has been widely accepted due to the small size and modest polarity of HMF and furfural. However, the hypersensitivity of *9xart*Δ mutants, where nutrient transporters accumulate at the plasma membrane, and the striking resistance of *10xaa*Δ strains lacking major amino acid permeases reveal that transporter-mediated uptake contributes substantially to intracellular accumulation of FUR and, at higher doses, HMF. Thus, our data support a dual-entry model in which passive diffusion provides a basal route, while surface transporters amplify toxic exposure when dysregulated. This framework reconciles historical diffusion-based models with modern transporter biology and suggests that similar transporter-dependent effects may be conserved across eukaryotes. Our results can also be used to contextualise previous efforts to understand toxic effects of furanic compounds, a common issue during conversion of lignocellulosic material for biofuel and chemical production (Jönsson et al., 2013). A CRISPR screen has previously been performed to identify yeast genes involved in tolerating lignocellulosic toxins, including the Ume6 transcriptional regulator and the Hog1 stress response gene (Gutmann et al., 2021). Ume6 has been shown to indirectly regulate nutrient transporter trafficking (Amoiradaki et al., 2021) and Hog1 activity is thought to correlate with expression of surface transporters in response to osmotic stress (Guo et al., 2026), further suggesting that toxicity could be regulated by active transport.

The intracellular targets of internalised furanic compounds are not well understood, but physiological effects reported in animal cell models have been proposed in the context of mitochondrial dysfunction (Qiu et al., 2022). To test this in yeast, we expressed a GFP-tagged version of Tom6, a component of the Translocase of the Outer Mitochondrial membrane complex (Pfanner et al., 1996). Under control conditions with DMSO, or even with addition of FUR and FMK at concentrations that inhibit growth, we observed no obvious defects in mitochondria morphology, even using 3D confocal microscopy. In contrast, addition of HMF induced fragmentation of mitochondria, with foci dispersed throughout the 3D volume of the cytoplasm (Figure7). This suggests furanic compounds drive increased mitochondrial fission, an adaptive stress response in eukaryotic cells associated with starvation and stress conditions that can lead to cell death (Pagliuso et al., 2018; Wang et al., 2022; Westermann, 2012). We also observed a decrease in mitochondrial activity following the addition of HMF. Thus, changes in mitochondrial morphology and activity in yeast may correlate with conserved oxidative stress and metabolic and inflammatory responses following furanic compound exposure in animal cells (Lee et al., 2019; Qiu et al., 2022; Shapla et al., 2018). These observations also align with research showing exposure of furanic compounds to different parasite species, including *Trypanosoma cruzi* and *Leishmania amazonensis*, inhibits growth (Hartmann et al., 2017; Sifontes-Rodríguez et al., 2015). Indeed, a recent study in the parasite *Toxoplasma gondii* showed growth defects and mitochondrial perturbations following exposure to furanic compounds (Portes et al., 2025). Curiously, no obvious mitochondrial defects of FUR and FMK were observed, at the concentrations tested. To further explore potential targets of these other furanic compounds, we considered the vacuole a potential target given the data implicating transporters and endolysosomal trafficking (Figure 5 & 6). Furthermore, the vacuole is associated with downregulation of transporters in response to other stresses, like different nutrient starvation conditions (Laidlaw et al., 2021; Müller et al., 2015; Paine et al., 2021). Although HMF had no effect on vacuolar morphology, FUR induced a series of mis-localisation phenotypes, that may be directly or indirectly related to the stress induced by FUR leading to cell death (Figure 8).

Although FMK concentrations that effectively inhibit yeast growth in a concentration manner were established, our genetic models did not identify conditions where cellular sensitivity or resistance to FMK were altered compared to wild-type controls, and FMK did not exhibit morphological defects in mitochondria or vacuoles. This suggests that FMK may provide toxicity by an entirely distinct mechanism, or a redundant combination of mechanisms. Less is known about FMK in the literature but this compound has already been identified in several thermally processed foods and can inhibit microbial driven production of biofuel (EFSA Panel et al., 2017; Guarnieri et al., 2017; Sayre et al., 1993). Beyond this, structurally related methylfuran compounds have been shown to form adducts with essential cytosolic proteins and perturb NAD^+^/NADH balance in yeast (Jilani & Olson, 2023), indicating FMK might similarly disrupt central metabolism rather than organellar integrity.

Having identified concentrations to study FMK in yeast, future work may provide insight as to its mode of action, which would be relevant for understanding toxic effects in humans. In conclusion, MRPs are complex and diverse, so concentrating our study on not just one but three furanic compounds allowed the specificity and modes of action to be compared. Strikingly, even across relatively similar compounds HMF, FUR and FMK, when studied in isolation we find each elicits differences: in effective concentrations, specificity for uptake/efflux, and perturbation of distinct organelles. The extensive complexity of these physiological responses will be difficult to unravel, but our work provides a framework to do so in model organisms that can reveal novel and relevant mechanistic models that can be tested in animal cell models. Beyond this, as such MRPs are inhibitory to biotechnological processes, such as the production of biofuel (Allen et al., 2010; Ask et al., 2013; Ren et al., 2024; Wang et al., 2016), this study can guide applications to modulate yeast tolerance for improved processing. Our findings contribute to a better understanding of how even relatively similar furanic compounds elicit different organelle-specific effects. This complexity that should be considered when trying to define the effects of MRPs in the context of human health. Elucidating these mechanisms in yeast not only facilitates screening for dietary safety thresholds but also aids in identifying biomarkers of exposure or damage that could be relevant for human toxicology. Mechanistic knowledge strengthens food risk assessment frameworks and supports evidence-based regulation of processing contaminants.

## Materials & Methods

### Yeast strains used in this study

Parental yeast strain BY4742 (Brachmann et al., 1998) was used for experimental work throughout the manuscript. BY4742 was the parental strain to test the role of Pdr5 (BY4742 *pdr5Δ::Kan^r^* (Giaever et al., 2002) and alpha arrestins (*9xart*Δ: BY4742 *ecm21*Δ *csr2*Δ *bsd2*Δ *rog3*Δ *rod1*Δ *ygr068c*Δ *aly2*Δ *aly1*Δ *ldb19*Δ *ylr392c*Δ (Nikko & Pelham, 2009). BY4741 (Brachmann et al., 1998) was the parent for the *NOP1*-GFP-Tom6 and *NOP1*-GFP-Vph1 strains for confocal microscopy (Weill et al., 2018; Yofe et al., 2016). To assess the role of yeast lacking nutrient transporters, the Σ22574d parental strain (Jauniaux & Grenson, 1990) was used following deletion of ten distinct transporters (*10xaa*Δ: *gap1*Δ *put4*Δ *uga4*Δ *can1*Δ *lyp1*Δ *apl1*Δ *hip1*Δ *dip5*Δ *gnp1*Δ *agp1*Δ (Besnard et al., 2016).

### Cell culture

Prior to culturing, yeast strains were grown on yeast extract peptone dextrose (YPD) agar medium plates (2% peptone, 2% glucose, 1% yeast extract, 2% agar) (Formedium, Norfolk, UK) overnight at 30°C. Yeast cultures were grown in synthetic complete (SC) medium (2% glucose, yeast nitrogen base supplemented with a complete mixture of required amino acid, bases and vitamins) (Formedium, Norfolk, UK) overnight at 30°C with shaking to early/mid-log phase (OD_600_ ≤ 1.0) prior to experimentation.

### Steady state growth assays

WT yeast cultures were grown overnight to saturation and 1.5 μl of culture was used to inoculate 200 μl SC medium with a stated titration of three furfural compounds (FC): 5-Hydroxymethylfurfural (HMF)), Furyl methyl ketone (FMK) or Furfual (FUR) (Sigma-Aldrich, USA) or Dimethyl sulfoxide (DMSO) was used as a control in a 96-well plate format. Plates were incubated at room temperature and subjected to readings at OD_600_ at 1hr and 24hrs (*pdr5Δ and 10xaa*Δ), and 1hr and 48hrs (*9xart*Δ) using a microplate spectrophotometer (ThermoMultiskan Go 1510 Sky, Thermo Fisher Scientific Inc., Massachusetts, US). Data was normalized to % cell viability, considering the average growth of each cell strain (without any exposure) as 100% and plotted in GraphPad Prism (version 8) to compare the statistical significance between experimental conditions, with *P*-values included. All end point assays are presented from at least 6 technical and 3 biological repeats. An asterisk (*) is used to denote significance *p* < 0.05.

### Continuous growth assays

96-well plates were set up following the same method as above. Growth experiments were monitored continuously for 24 hrs using a Stratus microplate reader (Cerillo, Charlottesville, VA, US) at 30°C. Growth curves presented are averages of at least six technical replicates.

### Confocal microscopy

GFP-labelled yeast strains were grown overnight to mid-log phase, cells were pelleted and resuspended in media supplemented with the three different FC compounds at stated concentrations. For experiments using fluorescent dyes, mid-log phase cells were first cultured prior to labelling. For mitochondria straining, 2 μM MitoTracker CMXRos (Thermo Fisher Scientific) was incubated for 30 minutes at 30°C, then washed. For vacuolar staining, 0.8 μM (*N*-(3-Triethylammoniumpropyl)-4-(6-(4-(Diethylamino) Phenyl) Hexatrienyl) Pyridinium Dibromide) FM4-64 (Thermo Fisher Scientific) and incubated for 30 minutes, followed by a 1-hour chase period in label free media. All media used for labelling, washing and chasing, for either MitoTracker or FM4-64, was supplemented with DMSO or stated furanic compound. Cells were imaged at room temperature using a 63x 1.40 oil immersion Plan Apochromat objective lens on a laser scanning confocal microscope (Zeiss LSM980). GFP fluorescence was excited using a 488 nm Argon laser and 495–550 nm emission collected. For cells labelled with red fluorescent dyes, imaging was performed at room temperature using a 63x 1.40 oil immersion Plan Apochromat objective lens on a Zeiss LSM 980 with Airyscan2. MitoTracker red and FM4-64 fluorescence was excited using a 561 nm Argon laser and 570-620 nm emissions collected. Processed images were adjusted in Fiji/ImageJ software (NIH).

### Statistical analyses

One-Way ANOVA followed by Sidak’s multiple comparisons test was utilized to compare wild-type (WT) cells with deletion strains under the same conditions to determine significant differences (p < 0.05). Additionally, an unpaired *t*-test was performed to compare normal organelles from GFP-exposed cells with control cells (GraphPad Prism v8, USA).

## Supplementary Material

Sup. Figure 1

Sup. Movie 1

Sup. Movie 2

## References

[R1] Agcam E (2022). A Kinetic Approach to Explain Hydroxymethylfurfural and Furfural Formations Induced by Maillard, Caramelization, and Ascorbic Acid Degradation Reactions in Fruit Juice-Based Mediums. Food Analytical Methods.

[R2] Allen SA, Clark W, McCaffery JM, Cai Z, Lanctot A, Slininger PJ, Liu ZL, Gorsich SW (2010). Furfural induces reactive oxygen species accumulation and cellular damage in Saccharomyces cerevisiae. Biotechnology for Biofuels.

[R3] Alsafra Z, Scholl G, Meulenaer BD, Eppe G, Saegerman C (2022). Hazard Ratio and Hazard Index as Preliminary Estimators Associated to the Presence of Furans and Alkylfurans in Belgian Foodstuffs. Foods.

[R4] Alvarez CE (2008). On the origins of arrestin and rhodopsin. BMC Evolutionary Biology.

[R5] Amoiradaki K, Bunting KR, Paine KM, Ayre JE, Hogg K, Laidlaw KME, MacDonald C (2021). The Rpd3-Complex Regulates Expression of Multiple Cell Surface Recycling Factors in Yeast. International Journal of Molecular Sciences.

[R6] Ask M, Bettiga M, Mapelli V, Olsson L (2013). The influence of HMF and furfural on redox-balance and energy-state of xylose-utilizing Saccharomyces cerevisiae. Biotechnology for Biofuels.

[R7] Aydin Y, Yilmaz B, Dikbasan YU, Orta-Yilmaz B (2023). Assessment of the oxidative damage and apoptotic pathway related to furan cytotoxicity in cultured mouse Leydig cells. Toxicology Research.

[R8] Balzi E, Wang M, Leterme S, Dyck LV, Goffeau A (1994). PDR5, a novel yeast multidrug resistance conferring transporter controlled by the transcription regulator PDR1. The Journal of Biological Chemistry.

[R9] Baril SA, Gose T, Schuetz JD (2023). How Cryo-EM Has Expanded Our Understanding of Membrane Transporters. Drug Metabolism and Disposition.

[R10] Başaran B, Çuvalcı B, Kaban G (2023). Dietary Acrylamide Exposure and Cancer Risk: A Systematic Approach to Human Epidemiological Studies. Foods.

[R11] Batool Z, Singla RK, Kamal MA, Shen B (2025). Demystifying furan formation in foods: Implications for human health, detection, and control measures: A review. Comprehensive Reviews in Food Science and Food Safety.

[R12] Batool Z, Xu D, Zhang X, Li X, Li Y, Chen Z, Li B, Li L (2021). A review on furan: Formation, analysis, occurrence, carcinogenicity, genotoxicity and reduction methods. Critical Reviews in Food Science and Nutrition.

[R13] Belle DV, André B (2001). A genomic view of yeast membrane transporters. Current Opinion in Cell Biology.

[R14] Besnard J, Pratelli R, Zhao C, Sonawala U, Collakova E, Pilot G, Okumoto S (2016). UMAMIT14 is an amino acid exporter involved in phloem unloading in Arabidopsis roots. Journal Of Experimental Botany.

[R15] Bianchi F, Klooster JS, van’t Ruiz, Poolman B (2019). Regulation of Amino Acid Transport in Saccharomyces cerevisiae. Microbiology and Molecular Biology Reviews.

[R16] Brachmann CB, Davies A, Cost GJ, Caputo E, Li J, Hieter P, Boeke JD (1998). Designer deletion strains derived from Saccharomyces cerevisiae S288C: a useful set of strains and plasmids for PCR-mediated gene disruption and other applications. Yeast (Chichester, England).

[R17] Chen X, Tu Q, Zhao W, Lin X, Chen Z, Li B, Zhang Y (2024). 5-Hydroxymethylfurfural mediated developmental toxicity in Drosophila melanogaster. Food and Chemical Toxicology.

[R18] Cheng Z, Luo X, Zhu Z, Huang Y, Yan X (2022). Furfural Produces Dose-Dependent Attenuating Effects on Ethanol-Induced Toxicity in the Liver. Frontiers in Pharmacology.

[R19] dos Conceição L, de Almeida BS, de Souza SF, Martinez VO, de Matos MFR, Andrade LL, Ruggiero WF, Matos LCP (2024). Critical conditions for the formation of Maillard Reaction Products (MRP) in bread: An integrative review. Journal of Cereal Science.

[R20] Cong K, Peng M, Kousholt AN, Lee WTC, Lee S, Nayak S, Krais J, VanderVere-Carozza PS, Pawelczak KS, Calvo J, Panzarino NJ (2021). Replication gaps are a key determinant of PARP inhibitor synthetic lethality with BRCA deficiency. Molecular Cell.

[R21] Donzella L, Sousa MJ, Morrissey JP (2023). Evolution and functional diversification of yeast sugar transporters. Essays in Biochemistry.

[R22] Knutsen HK, Alexander J, Barregård L, Bignami M, Brüschweiler B, Ceccatelli S, Cottrill B, Dinovi M, Edler L, Grasl-Kraupp B, EFSA_Panel (2017). Risks for public health related to the presence of furan and methylfurans in food. EFSA Journal.

[R23] Egner R, Rosenthal FE, Kralli A, Sanglard D, Kuchler K (1998). Genetic separation of FK506 susceptibility and drug transport in the yeast Pdr5 ATP-binding cassette multidrug resistance transporter. Molecular Biology of the Cell.

[R24] Eisenbrand G (2020). Revisiting the evidence for genotoxicity of acrylamide (AA), key to risk assessment of dietary AA exposure. Archives of Toxicology.

[R25] Gauthier-Coles G, Vennitti J, Zhang Z, Comb WC, Xing S, Javed K, Bröer A, Bröer S (2021). Quantitative modelling of amino acid transport and homeostasis in mammalian cells. Nature Communications.

[R26] Giaever G, Chu AM, Ni L, Connelly C, Riles L, Véronneau S, Dow S, Lucau-Danila A, Anderson K, André B, Arkin AP (2002). Functional profiling of the Saccharomyces cerevisiae genome. Nature.

[R27] Golin J, Ambudkar SV (2015). The multidrug transporter Pdr5 on the 25th anniversary of its discovery: an important model for the study of asymmetric ABC transporters. Biochemical Journal.

[R28] Golin J, Schmitt L (2023). Pdr5: A master of asymmetry. Drug Resistance Updates.

[R29] Guarnieri MT, Franden MA, Johnson CW, Beckham GT (2017). Conversion and assimilation of furfural and 5-(hydroxymethyl)furfural by Pseudomonas putida KT2440. Metabolic Engineering Communications.

[R30] Guo H, Wang Q, Lv W, Zhang Y, Wang F, Yuan Y, Yue T (2026). Dual-phase Hog1 activation and transporter gene reprogramming enable extreme sugar tolerance in food osmophilic yeasts. Food Microbiology.

[R31] Gutmann F, Jann C, Pereira F, Johansson A, Steinmetz LM, Patil KR (2021). CRISPRi screens reveal genes modulating yeast growth in lignocellulose hydrolysate. Biotechnology for Biofuels.

[R32] Hartmann AP, de Carvalho MR, Bernardes LSC, de Moraes MH, de Melo EB, Lopes CD, Steindel M, da Silva JS, Carvalho I (2017). Synthesis and 2D-QSAR studies of neolignan-based diaryl-tetrahydrofuran and -furan analogues with remarkable activity against Trypanosoma cruzi and assessment of the trypanothione reductase activity. European Journal of Medicinal Chemistry.

[R33] Heer D, Sauer U (2008). Identification of furfural as a key toxin in lignocellulosic hydrolysates and evolution of a tolerant yeast strain. Microbial Biotechnology.

[R34] Hosry LE, Elias V, Chamoun V, Halawi M, Cayot P, Nehme A, Bou-Maroun E (2025). Maillard Reaction: Mechanism, Influencing Parameters, Advantages, Disadvantages, and Food Industrial Applications: A Review. Foods.

[R35] Jauniaux J, Grenson M (1990). GAP1, the general amino acid permease gene of Saccharomyces cerevisiae. European Journal of Biochemistry.

[R36] Jilani SB, Olson DG (2023). Mechanism of furfural toxicity and metabolic strategies to engineer tolerance in microbial strains. Microbial Cell Factories.

[R37] Jönsson LJ, Alriksson B, Nilvebrant N-O (2013). Bioconversion of lignocellulose: inhibitors and detoxification. Biotechnology for Biofuels.

[R38] Kahlhofer J, Leon S, Teis D, Schmidt O (2021). The α-arrestin family of ubiquitin ligase adaptors links metabolism with selective endocytosis. Biology of the Cell.

[R39] Karapanagioti F, Atlason ÚÁ, Slotboom DJ, Poolman B, Obermaier S (2024). Fitness landscape of substrate-adaptive mutations in evolved APC transporters.

[R40] Kathuria D, Hamid Gautam S, Thakur A (2023). Maillard reaction in different food products: Effect on product quality, human health and mitigation strategies. Food Control.

[R41] Kell DB (2021). The Transporter-Mediated Cellular Uptake and Efflux of Pharmaceutical Drugs and Biotechnology Products: How and Why Phospholipid Bilayer Transport Is Negligible in Real Biomembranes. Molecules.

[R42] Kim D, Hahn JS (2013). Roles of the Yap1 Transcription Factor and Antioxidants in Saccharomyces cerevisiae’s Tolerance to Furfural and 5-Hydroxymethylfurfural, Which Function as Thiol-Reactive Electrophiles Generating Oxidative Stress. Applied and Environmental Microbiology.

[R43] Klinke HB, Thomsen AB, Ahring BK (2004). Inhibition of ethanol-producing yeast and bacteria by degradation products produced during pre-treatment of biomass. Applied Microbiology and Biotechnology.

[R44] Kohler V, Büttner S (2021). Remodelling of Nucleus-Vacuole Junctions During Metabolic and Proteostatic Stress. Contact.

[R45] Kong F, Lee BH, Wei K (2019). 5-Hydroxymethylfurfural Mitigates Lipopolysaccharide-Stimulated Inflammation via Suppression of MAPK, NF-κB and mTOR Activation in RAW 264.7 Cells. Molecules.

[R46] Laidlaw KME, Bisinski DD, Shashkova S, Paine KM, Veillon MA, Leake MC, MacDonald C (2021). A glucose-starvation response governs endocytic trafficking and eisosomal retention of surface cargoes in budding yeast. Journal of Cell Science.

[R47] Laidlaw KME, Calder G, MacDonald C (2022). Recycling of cell surface membrane proteins from yeast endosomes is regulated by ubiquitinated Ist1. The Journal of Cell Biology.

[R48] Laidlaw KME, MacDonald C (2018). Endosomal trafficking of yeast membrane proteins. Biochemical Society Transactions.

[R49] Laidlaw KME, Nadir HH, Milburn A, Xelhuantzi MSC, Stanislovas J, Droop AP, MacDonald S, Andreev I, Leech A, Ungar D, Sadhu MJ (2025). Killer toxin K28 resistance in yeast relies on COG complex-mediated trafficking of the defence factor Ktd1. Journal of Cell Science.

[R50] Lam FH, Turanlı-Yıldız B, Liu D, Resch MG, Fink GR, Stephanopoulos G (2021). Engineered yeast tolerance enables efficient production from toxified lignocellulosic feedstocks. Science Advances.

[R51] Lee C-H, Chen K-T, Lin J-A, Chen Y-T, Chen Y-A, Wu J-T, Hsieh C-W (2019). Recent advances in processing technology to reduce 5-hydroxymethylfurfural in foods. Trends in Food Science & Technology.

[R52] Leopardi P, Cordelli E, Villani P, Cremona TP, Conti L, Luca GD, Crebelli R (2010). Assessment of in vivo genotoxicity of the rodent carcinogen furan: evaluation of DNA damage and induction of micronuclei in mouse splenocytes. Mutagenesis.

[R53] Leppert G, McDevitt R, Falco SC, Dyk TKV, Ficke MB, Golin J (1990). Cloning by gene amplification of two loci conferring multiple drug resistance in Saccharomyces. Genetics.

[R54] Li SC, Kane PM (2009). The yeast lysosome-like vacuole: Endpoint and crossroads. Biochimica et Biophysica Acta (BBA) - Molecular Cell Research.

[R55] Lin CH, MacGurn JA, Chu T, Stefan CJ, Emr SD (2008). Arrestin-Related Ubiquitin-Ligase Adaptors Regulate Endocytosis and Protein Turnover at the Cell Surface. Cell.

[R56] Lin JH, Yamazaki M (2002). Role of P-glycoprotein in pharmacokinetics: clinical implications. Clinical Pharmacokinetics.

[R57] Liu S, Sun H, Ma G, Zhang T, Wang L, Pei H, Li X, Gao L (2022). Insights into flavor and key influencing factors of Maillard reaction products: A recent update. Frontiers in Nutrition.

[R58] MacDonald C, Piper RC (2016). Cell surface recycling in yeast: mechanisms and machineries. Biochemical Society Transactions.

[R59] MacDonald C, Piper RC (2017). Genetic dissection of early endosomal recycling highlights a TORC1-independent role for Rag GTPases. Journal of Cell Biology.

[R60] MacDonald C, Shields SB, Williams CA, Winistorfer S, Piper RC (2020). A Cycle of Ubiquitination Regulates Adaptor Function of the Nedd4-Family Ubiquitin Ligase Rsp5. Current Biology.

[R61] Megarioti AH, Primo C, Kapetanakis GC, Athanasopoulos A, Sophianopoulou V, André B, Gournas C (2021). The Bul1/2 Alpha-Arrestins Promote Ubiquitylation and Endocytosis of the Can1 Permease upon Cycloheximide-Induced TORC1-Hyperactivation. International Journal of Molecular Sciences.

[R62] Müller M, Schmidt O, Angelova M, Faserl K, Weys S, Kremser L, Pfaffenwimmer T, Dalik T, Kraft C, Trajanoski Z, Lindner H (2015). The coordinated action of the MVB pathway and autophagy ensures cell survival during starvation. eLife.

[R63] Neuwirth C, Mosesso P, Pepe G, Fiore M, Malfatti M, Turteltaub K, Dekant W, Mally A (2012). Furan carcinogenicity: DNA binding and genotoxicity of furan in rats in vivo. Molecular Nutrition & Food Research.

[R64] Nie S, Huang J, Hu J, Zhang Y, Wang S, Li C, Marcone M, Xie M (2013). Effect of pH, temperature and heating time on the formation of furan in sugar–glycine model systems. Food Science and Human Wellness.

[R65] Nikko E, Pelham HRB (2009). Arrestin-Mediated Endocytosis of Yeast Plasma Membrane Transporters. Traffic (Copenhagen, Denmark).

[R66] Nikko E, Sullivan JA, Pelham HRB (2008). Arrestin-like proteins mediate ubiquitination and endocytosis of the yeast metal transporter Smf1. EMBO Reports.

[R67] Nowak A, Janoszka B, Szumska M, Tyrpien-Golder K (2021). Furfural, Hydroxymethylfurfural and Furosine as Maillard Reaction Markers in Fruit Based Foods Including Jams and Baby Food. Journal of Microbiology, Biotechnology and Food Sciences.

[R68] O’Donnell AF, Schmidt MC (2019). AMPK-Mediated Regulation of Alpha-Arrestins and Protein Trafficking. International Journal of Molecular Sciences.

[R69] Pagare PP, McGinn M, Ghatge MS, Shekhar V, Alhashimi RT, Pierce BD, Abdulmalik O, Zhang Y, Safo MK (2024). The antisickling agent, 5-hydroxymethyl-2-furfural: Other potential pharmacological applications. Medicinal Research Reviews.

[R70] Pagliuso A, Cossart P, Stavru F (2018). The ever-growing complexity of the mitochondrial fission machinery. Cellular and Molecular Life Sciences.

[R71] Paine KM, Ecclestone GB, MacDonald C (2021). Fur4-mediated uracil-scavenging to screen for surface protein regulators. Traffic.

[R72] Pfanner N, Douglas MG, Endo T, Hoogenraad NJ, Jensen RE, Meijer M, Neupert W, Schatz G, Schmitz UK, Shore GC (1996). Uniform nomenclature for the protein transport machinery of the mitochondrial membranes. Trends in Biochemical Sciences.

[R73] Piecuch A, Obłąk E (2014). Yeast ABC proteins involved in multidrug resistance. Cellular & Molecular Biology Letters.

[R74] Piper RC, Katzmann DJ (2007). Biogenesis and Function of Multivesicular Bodies. Annual Review of Cell and Developmental Biology.

[R75] Poot M, Zhang YZ, Krämer JA, Wells KS, Jones LJ, Hanzel DK, Lugade AG, Singer VL, Haugland RP (1996). Analysis of mitochondrial morphology and function with novel fixable fluorescent stains. The Journal of Histochemistry and Cytochemistry : Official Journal of the Histochemistry Society.

[R76] Portes JdeA, Achari A, Vinayagam J, Bhattacharjee P, Chatterjee S, Jaisankar P, de Souza W (2025). Furan derivative affects the cell division and mitochondrial integrity of tachyzoites of Toxoplasma gondii. Experimental Parasitology.

[R77] Program NT (1993). Toxicology and Carcinogenesis Studies of Furan in F344 Rats and B6C3F1 Mice. National Toxicology Program Technical Report Series.

[R78] Program NT (2010). NTP toxicology and carcinogenesis studies of 5-(Hydroxymethyl)-2-furfural in F344/N rats and B6C3F1 mice. National Toxicology Program Technical Report Series.

[R79] Qiu Y, Lin X, Chen Z, Li B, Zhang Y (2022). 5-Hydroxymethylfurfural Exerts Negative Effects on Gastric Mucosal Epithelial Cells by Inducing Oxidative Stress, Apoptosis, and Tight Junction Disruption. Journal of Agricultural and Food Chemistry.

[R80] Quan W, Lin Y, Xue C, Cheng Y, Luo J, Lou A, Zeng M, He Z, Shen Q, Chen J (2022). Metabolic perturbations and health impact from exposure to a combination of multiple harmful Maillard reaction products on Sprague-Dawley rats. Food & Function.

[R81] Ren J, Zhang M, Guo X, Zhou X, Ding N, Lei C, Jia C, Wang Y, Zhao J, Dong Z, Lu D (2024). Furfural tolerance of mutant Saccharomyces cerevisiae selected via ionizing radiation combined with adaptive laboratory evolution. Biotechnology for Biofuels and Bioproducts.

[R82] Rogers B, Decottignies A, Kolaczkowski M, Carvajal E, Balzi E, Goffeau A (2001). The pleitropic drug ABC transporters from Saccharomyces cerevisiae. Journal of Molecular Microbiology and Biotechnology.

[R83] Santos LdeO, Ribeiro ALS, Lima KMdeO, dos Santos IRP, de Almeida BS, de Matos MFR, Krumreich FD, Andrade LL, Ruggiero WF, Matos LCP (2025). Critical factors associated with Maillard Reaction Products in different meats: an integrative review. Food Control.

[R84] Sayre LM, Arora PK, Iyer RS, Salomon RG (1993). Pyrrole formation from 4-hydroxynonenal and primary amines. Chemical Research in Toxicology.

[R85] Shakoor A, Zhang C, Xie J, Yang X (2022). Maillard reaction chemistry in formation of critical intermediates and flavour compounds and their antioxidant properties. Food Chemistry.

[R86] Shapla UM, Solayman Md, Alam N, Khalil MdI, Gan SH (2018). 5-Hydroxymethylfurfural (HMF) levels in honey and other food products: effects on bees and human health. Chemistry Central Journal.

[R87] Shaw JM, Nunnari J (2002). Mitochondrial dynamics and division in budding yeast. Trends in Cell Biology.

[R88] Sifontes-Rodríguez S, Monzote-Fidalgo L, Castañedo-Cancio N, Montalvo-Álvarez AM, López-Hernández Y, Diogo NM, Infante-Bourzac JF, Pérez-Martín O, Meneses-Marcel A, García-Trevijano JAE, Cabrera-Pérez MÁ (2015). The efficacy of 2-nitrovinylfuran derivatives againstLeishmania in vitro and in vivo. Memórias Do Instituto Oswaldo Cruz.

[R89] Sodani K, Patel A, Kathawala RJ, Chen ZS (2012). Multidrug resistance associated proteins in multidrug resistance. Chinese Journal of Cancer.

[R90] Sun G, Wang P, Chen W, Hu X, Chen F, Zhu Y (2022). Simultaneous quantitation of acrylamide, 5-hydroxymethylfurfural, and 2-amino-1-methyl-6-phenylimidazo[4,5-b]pyridine using UPLC-MS/MS. Food Chemistry.

[R91] Tamanna N, Mahmood N (2015). Food Processing and Maillard Reaction Products: Effect on Human Health and Nutrition. International Journal of Food Science.

[R92] Teixeira MC, Dias PJ, Simões T, Sá-Correia I (2008). Yeast adaptation to mancozeb involves the up-regulation of FLR1 under the coordinate control of Yap1, Rpn4, Pdr3, and Yrr1. Biochemical and Biophysical Research Communications.

[R93] Wang S, Cheng G, Joshua C, He Z, Sun X, Li R, Liu L, Yuan Q (2016). Furfural tolerance and detoxification mechanism in Candida tropicalis. Biotechnology for Biofuels.

[R94] Wang S, Tan J, Miao Y, Zhang Q (2022). Mitochondrial Dynamics, Mitophagy, and Mitochondria–Endoplasmic Reticulum Contact Sites Crosstalk Under Hypoxia. Frontiers in Cell and Developmental Biology.

[R95] Weill U, Yofe I, Sass E, Stynen B, Davidi D, Natarajan J, Ben-Menachem R, Avihou Z, Goldman O, Harpaz N, Chuartzman S (2018). Genome-wide SWAp-Tag yeast libraries for proteome exploration. Nature Methods.

[R96] Westermann B (2012). Bioenergetic role of mitochondrial fusion and fission. Biochimica et Biophysica Acta (BBA) - Bioenergetics.

[R97] Wierckx N, Koopman F, Ruijssenaars HJ, de JH (2011). Microbial degradation of furanic compounds: biochemistry, genetics, and impact. Applied Microbiology and Biotechnology.

[R98] Yilmaz B, Aydin Y, Orta-Yilmaz B (2023). Furan promotes cytotoxic effects through DNA damage and cell apoptosis in Leydig cells. Toxicology Mechanisms and Methods.

[R99] Yofe I, Weill U, Meurer M, Chuartzman S, Zalckvar E, Goldman O, Ben-Dor S, Schütze C, Wiedemann N, Knop M, Khmelinskii A (2016). One library to make them all: streamlining the creation of yeast libraries via a SWAp-Tag strategy. Nature Methods.

[R100] Zbieralski K, Wawrzycka D (2022). α-Arrestins and Their Functions: From Yeast to Human Health. International Journal of Molecular Sciences.

[R101] Zeng R, Zhang G, Zheng J, Zhou H, Wang Y, Huang C, Hu W, Ou S (2020). Formation and Identification of Two Hydroxmethylfurfural–Glycine Adducts and Their Cytotoxicity and Absorption in Caco-2 Cells. Journal of Agricultural and Food Chemistry.

[R102] Zhang G-L, Liang Y, Zhu J-Y, Jia Q, Gan W-Q, Sun L-M, Hou H-M (2015). Oxidative stress-mediated antiproliferative effects of furan-containing sulfur flavors in human leukemia Jurkat cells. Food Chemistry.

[R103] Zhao Q, Zou Y, Huang C, Lan P, Zheng J, Ou S (2017). Formation of a Hydroxymethylfurfural–Cysteine Adduct and Its Absorption and Cytotoxicity in Caco-2 Cells. Journal of Agricultural and Food Chemistry.

[R104] Zhu Z, Fang R, Huang M, Wei Y, Zhou G (2020). Oxidation combined with Maillard reaction induced free and protein-bound Nε-carboxymethyllysine and Nε-carboxyethyllysine formation during braised chicken processing. Food Science and Human Wellness.

